# Endogenous Retroviruses as Targets for Antitumor Immunity in Renal Cell Cancer and Other Tumors

**DOI:** 10.3389/fonc.2013.00243

**Published:** 2013-09-17

**Authors:** Elena Cherkasova, Quinn Weisman, Richard W. Childs

**Affiliations:** ^1^Hematology Branch, National Heart, Lung and Blood Institute, National Institutes of Health, Bethesda, MD, USA

**Keywords:** human endogenous retroviruses, antigen, cytotoxic T-cells, cancer treatment, immunotherapy

## Abstract

Human endogenous retroviruses (HERVs), remnants of ancient germ-line infections with exogenous retroviruses, are estimated to comprise up to 8% of human genome. Most HERVs have accumulated mutations and deletions that prevent their expression as an infectious virus. Nevertheless, a growing number of HERV genes and proteins have been found to be expressed in different cancers, raising the possibility that HERV-derived antigens might represent excellent targets for tumor immunotherapy. Here, we review data showing HERV-encoded antigens are capable of eliciting humoral and T-cells specific antitumor immunity. We also describe a novel HERV-E that was recently found to be selectively expressed in over 80% of clear cell kidney cancer but not in normal tissues. Remarkably, the restricted expression of HERV-E in kidney tumors was found to occur as a consequence of inactivation of the von Hippel–Lindau tumor suppressor. Importantly, antigens derived from this provirus are immunogenic, stimulating cytotoxic T-cells that kill kidney cancer cells *in vitro* and *in vivo*. Taken altogether, these data suggest efforts aimed at boosting human immunity against HERV-derived antigens could be used as a strategy to treat advanced tumors including kidney cancer.

## Introduction

The basis for safe and effective adoptive cell therapy is to identify truly tumor-specific targets to minimize the risk of autoimmunity (1). Many tumor-associated antigens such as the melanoma antigens gp100 and MART-1 have already established their ability to induce antitumor T-cell immunity in clinical studies. Patients with melanoma have also been treated with T-cells engineered using recombinant retroviral vectors to express HLA-2 restricted high affinity T-cell receptors specific for these melanoma antigens (2, 3). Since the vast majority of antigens targeted to date are overexpressed self-antigens, there exists a need to overcome self-tolerance mechanisms that limit immune responses against these tumor targets. In contrast, viral antigens are known to induce strong antitumor immunity due to their foreign nature, although at present only a minority of human cancers has been shown to have a viral etiology and to express viral antigens. Over the last decade, there is a growing list of evidence indicating that human endogenous retrovirus (HERVs), which under normal conditions are silenced, can be activated in cancer cells, thus providing a new source of virus-derived antigens to serve as targets for cancer immunotherapy.

The vast majority of HERVs have accumulated mutations or deletions and thus do not contain uninterrupted ORFs to code full-length proteins, or have had their transcription silenced by promoter methylation. Nevertheless, a number of different HERVs have been shown to be transcriptionally active in human malignancies including but not limited to melanoma (4–6), breast cancer (7), prostate adenocarcinoma (8), ovarian cancer (9, 10), and most recently renal cell carcinoma (11, 12).

Metastatic kidney cancer appears to be susceptible to immune-based therapy and allogeneic immunotherapy (13, 14), but surprisingly little data exists characterizing the antigens expressed on the tumor cells targeted by immune cells that mediate regression of metastatic renal cancer. Recently, we isolated and expanded a CD8+ T-cell (CTL) clone from the blood of a patient with regressing renal cell carcinoma following an allogeneic hematopoietic stem cell transplant that killed patient tumor cells *in vitro* (11). This CTL clone was found to have tumor-specific cytotoxicity, recognizing an HLA-A11-restricted 10-mer peptide named CT-RCC-1. The transcripts encoding this antigen were found to be derived from a novel HERV-E (named CT-RCC HERV-E) located on chromosome 6q. At present, three transcripts (named CT-RCC-8, CT-RCC-9, and CT-RCC-Env) originating from this provirus have been discovered that encode for parts of the protease and polymerase as well as the entire envelope genes, respectively (GenBank accession numbers EU137846.1, EU137847.1, and JQ733905). Remarkably, all transcripts derived from the CT-RCC HERV-E were found to be selectively expressed in the clear cell variant of renal cell carcinoma (ccRCC), with no expression observed in normal tissues or any other type of tumor cells (12, 15). Importantly, since proviral expression has been detected in fresh tumors even at the earliest stages of disease, antigens derived from CT-RCC HERV-E could theoretically serve as ideal targets for T-cell based immunotherapy for kidney cancer.

Besides the HERV-E virus described above, which appears to elicit antitumor immunity against kidney cancer cells, there exists a growing number of studies showing other endogenous retroviral products can induce antitumor immunity in other tumors. This review summarizes and critically discusses available data on HERV-encoded antigens which potentially could serve as targets for future cancer immunotherapeutic approaches.

## HERV Reactivation in Cancer

Human endogenous retroviruses, which represent remnants of ancient retroviral infections, constitute about 8% of the human genome. Because approximately 80% of all CpG dinucleotides are methylated in the genome of differentiated somatic human cells, proviral LTRs are efficiently silenced by CpG methylation (16, 17). Abnormal hypomethylation of CpG dinucleotides is a known characteristic of many cancers. As a consequence, growing evidence suggests reactivation of HERVs can occur in a variety of cancers as the result of demethylated LTRs, which function as promoters, liberating HERV expression (9, 18–22).

Despite the fact that HERVs have been shown to be transcriptionally active in human malignancies, the mechanisms leading to abnormal cancer-specific activation of HERVs still remain mostly unclear. Early studies have suggested that expression of ubiquitous transcription factors such as Sp1, Myb, and YY1 proteins can stimulate transcription of HERVs from hypomethylated LTRs that contain essential binding sites (23–25). Recent data corroborated these observations by providing evidence that the HERV-K promoter activity is dependent on Sp1 and Sp3 transcription factors (26). Using chromatin immunoprecipitation (ChIP) and RNA interference assays, the binding of transcription factors Sp1 and Sp3 to the examined LTR promoter region of HERV-K was shown. These findings, however, have not revealed the essential mechanism accounting for the restricted expression of HERVs in specific types of cancer cells.

Other studies have focused on the mechanisms of HERV transcriptional control indicated the involvement of cell-type-specific transcription factor in proviral activation. In melanoma, the arrangement of the TATA-box, binding and initiator sites, and enhancer sequences in the HERV-K proviral LTR have been shown to play an important role in the reactivation of the provirus via binding of melanoma-specific isoform of microphthalmia-associated transcription factor (MITF-M) to the proviral LTR (27). In non-melanoma HEK293 cells, both chromosomal HERV-K expression and the cloned LTR function were strongly activated by forced expression of MITF-M. The requirement of MITF-M for the LTR activation seemed to provide a mechanism for melanoma-specific expression of HERV-K. In addition, expression of melanocyte-specific genes was also reported in breast cancers (28), which might also account for HERV-K expression in this cancer cell type.

**Figure 1:**
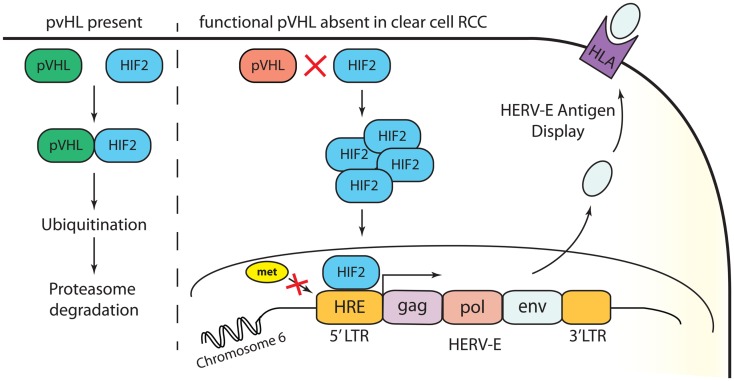
**Transcriptional up-regulation of CT-RCC HERV-E in clear cell RCC appears to be a consequence of three events: (i) *VHL* inactivation, (ii) HIF-2α overexpression, and (iii) hypomethylation of the HERV-E 5′LTR**.

Our recent studies have provided insights into the mechanisms accounting for the restricted expression of a HERV-E in the most common subtype of renal cell carcinoma, clear cell RCC (12). Clear cell carcinoma accounts for approximately 80% of all RCC tumors. In most clear cell cancers, the von Hippel–Lindau (*VHL*) tumor suppressor gene is inactivated from either a genetic mutation or promoter hypermethylation. We found that transcriptional up-regulation of HERV-E in ccRCC is associated with inactivation of the *VHL* gene. Using quantitative RT-PCR we found up to 90% of primary frozen ccRCC tumors obtained from nephrectomy samples from patients whose tumors had *VHL* mutations expressed HERV-E transcripts at high levels (in a range of 400 up to 25,000 copies relative to GAPDH × 10^5^). In addition, expression of CT-RCC HERV-E was reduced substantially by introducing wild type *VHL* transgenes into *VHL*-deficient ccRCC cell lines. It has been previously shown that hypoxia-inducible transcription factors (HIF) are stabilized in a vast majority of ccRCC tumors as a consequence of *VHL* inactivation (29). Using a ChIP assay, we demonstrated that the transcriptional factor HIF-2α plays a role in promoting expression of HERV-E by binding with a HIF response element (HRE) localized in its 5′LTR. Remarkably, we found the HRE motif as well as other surrounding CpGs in the 5′LTR were hypomethylated in all HERV-E expressing ccRCCs. In contrast, other types of tumors and normal tissues as well as the small subset of ccRCC cell lines that were found to be HERV-E negative, possessed a hypermethylated 5′LTR. Consistent with this observation, we found treating ccRCC cells that contain hypermethylated 5′LTR with the demethylating agent DAC and/or the histone deacetylase inhibitor depsipeptide increased HERV-E expression. In summary, these findings show the transcriptional up-regulation of HERV-E in ccRCC appears to be related to three critical events: (i) *VHL* inactivation, (ii) HIF-2α overexpression, and (iii) hypomethylation of the HERV-E 5′LTR (Figure 1).

The presence of multiple binding sites in retroviral LTRs contributes to the complexity and flexibility of HERV transcriptional activation. Additional studies are necessary to further reveal the exact cellular transcriptional regulatory mechanisms that allow cancer-specific expression of HERV loci in different types of tumors.

## Serological Response to HERVs in Cancer

Although most HERVs have extensive deletions and mutations that render them inactive, some have retained ORFs coding for full or partial proteins that potentially can be translated if the provirus is reactivated. For example, HERV-K, the youngest HERV from an evolutionary perspective, in some cases contains nearly intact ORFs with a few or even no mutations (30–32). The first studies that aimed to investigate a potential patho-physiological role of HERV-K revealed the presence of HERV-K specific antibodies in patients with germ cell tumors (33, 34). Sequences of intact *gag* and *env* ORFs in the HERV-K10 provirus were incorporated into a baculovirus vector; subsequently the recombinant proteins were then utilized to screen human serum for antibody reactivity by employing immunofluorescence. These studies revealed patients with seminoma or mixed germ cell tumors produced antibodies against Gag or Env proteins at a very high frequency. In contrast, healthy individuals and patients with different types of tumors rarely produced these antibodies.

In addition to these findings, a high prevalence of antibodies against HERV-K proteins was also found in the serum of patients with melanoma. Of note, the level of antibodies against HERV-K Env was shown to be significantly higher in sera from melanoma patients compared to that of normal blood donors (5, 35). Data from subsequent studies reported antibody responses against HERV-K proteins in melanoma patients was associated with poorer survival, and thus could potentially be used as a disease specific prognostic factor (36).

More recently, various studies have demonstrated that HERV-K is reactivated in the majority of human breast tumors. Significant titers of anti-HERV-K antibodies were detected in a high proportion of patients with breast cancer, but not in the serum of normal donors (37–39). Preclinical data further suggest proteins products of HERV-K could potentially serve as targets for antibody based treatments for breast cancer. In one study, immunodeficient mice were injected with human breast cancer cells and then subsequently infused with anti-HERV-K Env monoclonal antibodies (mAbs), which markedly reduced tumor development (40). *In vitro* results from this study showed anti-HERV-K Env mAb blocked both the growth and proliferation of human breast cancer cells.

Interestingly, in contrast to most cancers, three different HERV families (HERV-K, HERV-E, and ERV3) were found to be expressed in ovarian cancer (10). Remarkably, antibodies against HERV Env proteins, including anti-ERV3 (30%), anti-HERV-E (40%), and anti-HERV-K (55%), were detected in the serum of patients with ovarian cancer, but not in healthy controls. Taken altogether, these findings illustrate that expression of HERVs in cancer cells may trigger antigen-specific immune responses. Based on preclinical animal experiments, these data suggest mAbs targeting HERV antigens could potentially be used as novel immunotherapeutic agents.

## CTL Recognition of HERV Antigens

In a variety of tumors, the immunogenicity of HERV-originated proteins or peptides has been assessed by their ability to elicit CTL-mediated immune recognition. For example, in melanoma, HERV-encoded peptides expressed in the tumor have been shown to trigger antitumor CTL responses (41). A HERV-K-encoded HLA-A2-restricted peptide (termed HERV-K-Mel) that was recognized by CTLs on autologous tumor cells of a melanoma patient was identified utilizing a cDNA expression cloning. This peptide was found to originate from a very short ORF from a HML-6 provirus located on chromosome 16 encoding a HERV-K-MEL *env* transcript. This observation highlights the fact that antigenic peptides can be translated from small alternative ORFs within retroviral transcripts, even when a main ORF encoding for a retroviral protein is absent as a consequence of mutations. HERV-K-MEL was not found to be expressed in normal tissues, with the exception of testis, but was found in nearly 85% of melanoma samples. Besides melanoma, most tumor types did not express the HERV-K-MEL, apart from a few sarcoma and carcinoma samples which only rarely expressed this transcript. These observations suggest HERV-K-MEL may be a useful source of tumor-specific antigens that could be used for therapeutic vaccination for the majority of patients with melanoma as well as a small proportion of patients with carcinoma.

In other studies, CTLs against HERV-K Gag-derived peptides have been detected in patients with seminoma tumors (42). Based on previous reports that demonstrated an antibody response specific to the Gag protein of HERV-K10 (33), seminoma patients were screened for T-cell responses specific for HERV-K Gag protein derived peptides. Twenty-six subjects with a history of seminoma were recruited for this study and all met the following criteria: the seminoma had been cured with orchiectomy, followed by radiation therapy within 1 month of diagnosis, and showed no recurrence or metastases. Eighteen healthy donors with no history of germ cell tumors were also used in this study. The time range from treatment of the cancer to the blood donation for detection of anti-HERV T-cell responses varied from 6 months to 25 years. Four peptide pools representing potential epitopes of the Gag protein were used for an initial screening to detect T-cell responses in the patients that were previously diagnosed with seminoma. The pools consisted of peptides that were predicted to have a high binding affinity for relatively common HLA alleles. Remarkably, T-cells reactive to the HERV-K peptide pools were found in the PBMCs of seminoma patients at a much higher frequency compared to healthy controls where T-cell responses were rarely seen.

Just as HERV specific T-cells were observed in melanoma and seminoma patients, the ability of HERV-H Xp22.3 envelope peptides to induce strong tumor-specific T-cell responses was shown to occur in patients with gastrointestinal cancer (43). Previous studies found that HERV-H Xp22.3 *env* mRNA was expressed in gastrointestinal cancer, but not in other cancers or normal tissues (20). As a result, peptides predicted to bind to HLA-A*0201 derived from a putative 273 aa protein encoded from HERV-H Xp22.3 *env* ORF were used to stimulate peripheral blood T-cells to generate peptide specific CTLs (43). Remarkably, these CTLs induced tumor-specific lysis of gastrointestinal cancer cell lines that endogenously expressed the HERV-H Xp22.3 *env* ORF. Taken altogether, these findings demonstrate that the Env protein is synthesized, processed by proteasomal pathways, and presented in the context of HLA-A2.1 on the cancer cell surface.

In our laboratory, we isolated and expanded a CTL clone from the blood of a patient with regressing renal cell carcinoma following an allogeneic stem cell transplant that killed patient tumor cells *in vitro* (11). This CTL clone was found to have tumor-specific cytotoxicity, recognizing an HLA-A11-restricted antigen called CT-RCC-1 that originated from CT-RCC HERV-E. Because only about 15–18% of the human population possesses HLA-A11, immunotherapy approaches targeting the CT-RCC-1 antigen through tumor peptide vaccination would be limited to only a minority of patients with metastatic kidney cancer. However, it is possible that other immunogenic peptides derived from this HERV-E could be expressed on more common HLA class I molecules, a finding that potentially could broaden the application of immunotherapy approaches targeting antigens derived from this HERV to a greater percentage of patients with metastatic RCC. Most recently, we have observed transcripts encoding for the entire *env* gene of CT-RCC HERV-E that are also selectively expressed in a majority of ccRCC tumors, concurrent with two previously identified transcripts, with no expression observed in any other tumors or normal tissues (15). Analysis of the envelope transcript’s encoding capacities showed long ORFs potentially leading to translation of surface (SU) and transmembrane (TM) envelope proteins. Using a similar approach as was done with the HERV-H Xp22.3 Env, we generated HERV-E Env peptides derived from the SU and TM ORFs that were predicted to have a high binding affinity for HLA-A*0201. Monocyte derived dendritic cells of healthy HLA-A*0201+ donors were pulsed with these peptides to stimulate autologous T-cells *in vitro*. Several of these peptides were found to be immunogenic *in vitro*, stimulating CTLs that recognized HERV-E expressing ccRCC tumor cells transfected to express HLA-A*0201, but not wild type ccRCC that was HERV-E+/HLA-A*0201-negative (15). Since CT-RCC HERV-E sequences are not expressed in normal tissues, tolerance induction by T-cells, which would normally occur against self-antigens, would theoretically be less likely to occur. Therefore, CT-RCC Env antigens, when expressed in a newly formed ccRCC tumor, could represent excellent potential targets for T-cell based immunotherapy for kidney cancer. However, at present, the ability of the human immune system to recognize these HERV-E derived antigens has not been fully analyzed nor has the proposal that that avoidance of tolerance induction to these apparently tumor-specific antigens been tested experimentally. Despite these limitations, preliminary data suggest, the field of cancer immunotherapy could potentially be advanced by the identification of additional antigens derived from HERVs that are specifically expressed in tumor cells and are recognized by CTLs.

## Conclusion

Cancer remains a significant problem worldwide. Expression of potentially immunogenic HERV proteins that behave as tumor-associated antigens have been detected by a growing number of independent investigators in a variety of different types of tumors over the last decade. Similar to antiviral vaccines now used to prevent cervical cancer (anti-HPV vaccine) or hepatocellular carcinoma (anti-hepatitis B vaccine), vaccines against HERV antigens that are typically not expressed in normal tissues could stimulate long lasting CTL responses which might prevent or eradicate malignancies at their earliest stages or more advanced cancers where metastasis express proviral antigens. The capacity of HERVs to produce full or partial length proteins in different cancers and their capability to induce cellular and/or humoral immune responses in the host remains an exciting and open area of investigation which holds great therapeutic promise for the prevention and treatment of cancer.

## Conflict of Interest Statement

The authors declare that the research was conducted in the absence of any commercial or financial relationships that could be construed as a potential conflict of interest.
